# Development of an Emulgel for the Effective Treatment of Atopic Dermatitis: Biocompatibility and Clinical Investigation

**DOI:** 10.3390/gels10060370

**Published:** 2024-05-27

**Authors:** Almudena Gómez-Farto, Ana Leticia Jiménez-Escobar, Noelia Pérez-González, Herminia Castán, Beatriz Clares, Salvador Arias-Santiago, Trinidad Montero-Vílchez

**Affiliations:** 1 Instituto de Investigación Biotecnológica, Farmacéutica y Medicamentos Huérfanos, S.L, 18016 Granada, Spain; almudenagomez@invesbiofarm.com (A.G.-F.); anajimenez@invesbiofarm.com (A.L.J.-E.); clinica@invesbiofarm.com (N.P.-G.); ensayos@invesbiofarm.com (H.C.); 2 Department of Pharmacy & Pharmaceutical Technology, Faculty of Pharmacy, University of Granada, 18071 Granada, Spain; 3 Institute of Nanoscience and Nanotechnology (IN2UB), University of Barcelona, 08028 Barcelona, Spain; 4 Biosanitary Institute of Granada (ibs.GRANADA), 18012 Granada, Spain; salvadorarias@ugr.es (S.A.-S.); tmonterov@ugr.es (T.M.-V.); 5 Department of Dermatology, Granada School of Medicine, Granada University, Virgen de las Nieves University Hospital, 18012 Granada, Spain

**Keywords:** emulgel, atopic dermatitis, hyaluronic acid, EGF

## Abstract

Atopic dermatitis (AD) is a common dermatological disease affecting both children and adults. No drug-free emulgel has been developed and studied in vitro and in vivo for the treatment of AD. The aim of this study was to develop and assess the efficacy of a topical emulgel containing hyaluronic acid, glycerol, Calendula officinalis, Aloe vera, polyphenols and EGF for the concomitant treatment in patients with AD aged over 14. Objective skin barrier function parameters were included, such as transepidermal water loss (TEWL), skin temperature, pH, stratum corneum hydration, skin elasticity and erythema. The subjective opinion of the patients was determined including acceptability, absorption, comfort of use and tolerability, as well as the degree of improvement in patients’ quality of life. We observed an improvement in the subjective parameters studied and statistically significant differences in the objective parameters. Specifically, we found an improvement in TEWL (*p* = 0.006), erythema (*p* = 0.008) and hydration (*p* < 0.001), parameters indicating an improvement in the epidermal barrier. One hundred per cent of patients were satisfied with the product. Therefore, these results suggest that the product may contribute to the treatment of AD.

## 1. Introduction

The skin is the largest organ in the body, accounting for approximately 15% of an adult’s total body weight [1]. It performs four main functions: sensation, thermoregulation, protection, and acting as a barrier against infection, chemical stress, thermal stress, transcutaneous evaporative water loss and UV light and metabolism, through the production of vitamin D and lipid storage [2,3].

Atopic dermatitis (AD), also known as atopic eczema, is a chronic inflammatory skin disorder affecting approximately 13% of children and 7% of adults [4,5]. It is considered as the third most prevalent dermatological disease [6]. It is characterized by erythematous, scaly and intense pruritus with lesions located over the flexural surfaces [6,7], with pruritus being the hallmark symptom with a major impact on quality of life [8]. It is associated with an increased risk of allergic conditions, such as asthma and rhinitis, and with the opportunistic *Candida albicans* infection [9]. This cutaneous disorder is characterised by a breakdown of the skin barrier due to an impairment of the stratum corneum resulting from a diminution in lipids. The stratum corneum lipids retain water and act as a permeability barrier [10]. Due to this barrier dysfunction, the permeability and the antimicrobial function are compromised, colonization by *Staphylococcus aureus* being a common feature of AD, as well as secondary infections [3].

The diagnosis of AD is made clinically based on family history, morphology, the distribution of skin lesions and associated clinical signs. However, skin biopsy or other tests may be required to exclude other diseases [11]. To facilitate classification, the American Academy of Dermatology produced a new revision in 2003 of the Hanifin and Rajka criteria [12] which, although not validated, are considered suitable for use in clinical practice in the diagnosis of infants, children and adults [13].

Once the diagnosis has been made, it is necessary to determine the severity of the disease. The classic method is the AD score (SCORAD) developed by the European Task Force on AD (ETFAD).

Topical treatments are sufficient for mild to moderate AD management [14]. Current therapies are focused on reducing inflammation, restoring the skin barrier, and antibacterial therapy [14]. Local corticosteroids are used as a first-line treatment to treat moderate to severe AD, but long-term use will lead to telangiectasia, skin thinning, epidermal atrophy, and other adverse effects [14,15]. Daily moisturization is one of the cornerstones of AD treatment [16].

Emulgels are emulsion-based gels containing a gelling agent such as Carbopol 940, which combine the properties of emulsions and gels, such as easy spreadability, emollience, longer shelflife and enhanced skin penetration [17,18]. Emulgels present a high viscosity, which causes greater adherence to the skin, increasing the staying potential of the formulation on the skin [19]. Gels are a system composed of a network of cross-linked polymers in a liquid phase, while emulsions are mixtures of oil and water stabilized by emulsifying agents. This allows them to include in the composition both oil- and water-soluble ingredients and to create a sustained release delivery system by entrapping components in the gel network [18,20]. This makes emulgel a viable option for delivering botanical actives as a vehicle for their extracts [20], as well as a wide range of active ingredients such as moisturizers and anti-inflammatory agents [18].

Hyaluronic acid (HA) is a natural polysaccharide found in various biological components such as articular cartilage, synovial fluid, eye’s vitreous humour and skin [21,22]. The skin contains 50% of the total HA, and in the epidermis it is involved in the formation of a competent barrier [22]. It has a high biocompatibility, gelling capacity, viscoelasticity and mucoadhesiveness, as well as bioactivities such as anti-inflammatory and wound-healing effects [21]. Its viscoelasticity is due to the negative charges in the molecule, which allow it to retain a large amount of water, up to 70% of its weight, due to its remarkable hydrophilicity [22]. HA is also involved in cell proliferation and differentiation, and in stimulating cell movement [23].

Epidermal growth factor (EGF) plays an important role in the proliferation, differentiation and migration of different cell types, particularly epithelial cells [24]. EGF is important for keratinocyte proliferation and migration during re-epithelisation, associated with skin wound healing [23]. It also acts on fibroblasts, which are mainly responsible for the formation of the extracellular matrix by synthesising HA, collagen, elastin, etc. It induces angiogenesis (formation of new blood vessels) and mediates the inflammatory response. All this leads to tissue repair [25]. EGF also increases HA production in keratinocytes [23,26]. The EGF effect on keratinocytes may contribute to a protective role in AD pathogenesis [27], and topical application of EGF improved skin lesion severity, skin thickness, itching, serum total IgE level and TEWL in an induced AD mice model [28]. GFs loaded into nanoparticles of hyaluronan are released in a controlled manner, promoting wound healing and enhancing pharmacological effects [29].

Glycerol acts as humectant relieving clinical signs of dryness, such as scaling, and may help to reduce transepidermal water loss (TEWL). It diffuses into the stratum corneum and increases the water-holding capacity of a normal stratum corneum [30]. Glycerol can reduce interleukin 4 expression, which may affect AD disease and will enhance stratum corneum hydration [31].

Grape seed oil is rich in phenolic compounds such as flavonoids, carotenoids, tannins, and stilbenes. Its polyphenols can inhibit the release of arachidonic acid, which is responsible for the production of leukotrienes and prostaglandins, which activate the inflammatory response [32]. Phenolic compounds are potent antioxidants with a wide range of biological properties due to their molecular structure, as they can alleviate symptoms and inhibit the development of various skin disorders [33].

*Calendula officinalis* has antifungal and antimicrobial properties against a wide range of Gram-positive and Gram-negative bacteria. It also has angiogenic and fibroblastic properties, which have a positive effect on cell proliferation during the healing process [34].

*Aloe vera* has anti-inflammatory, antioxidant, anticancer, and wound-healing properties. When applied topically, it has shown inhibitory effects on AD symptoms and IgE levels [35].

Our formula includes an innovative patented method for stabilising EGF in the oily phase. Other drug-based emulgels have been developed for the treatment of AD [36,37], but no drug-free emulgel has yet been developed and studied in vivo or in vitro for the treatment of AD. This study aimed to develop and assess an emulgel containing HA, glycerol, *Calendula officinalis*, *Aloe vera*, polyphenols and EGF for AD concomitant treatment.

## 2. Results and Discussion

### 2.1. Emulgel Biocompatibility

The experiments carried out on the developed emulgel concluded that the formulation was biocompatible and safe to use.

#### 2.1.1. Skin Irritation

No erythema and oedema formations were observed at any of the application sites and injection points as showed in Table 1.

According to the data obtained from the observations and the defined evaluation criteria, the tested sample does not cause a skin irritation effect.

#### 2.1.2. Sensitization Test

No visible skin reactions were observed at the application sites of the test product extract. There was no discrete weight loss in the test animals. There were also no visible changes in the general health status of the animals.

According to the results of the observations and the evaluation criteria showed in Table 2, the tested product does not have a sensitizing effect (to material).

#### 2.1.3. Cytotoxicity Test

**Figure 1:**
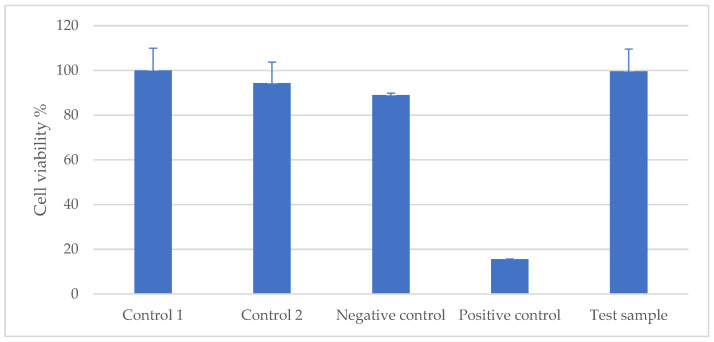
Percentage of cell viability of the test sample compared to the control sample (L929 mouse cell line).

According to the evaluation criteria given, the cell viability of the test item was quantitatively calculated in comparison with the control sample, as described in Figure 1. The cell viability was 99.62 ± 9.97%, so it can be concluded that the test item has no cytotoxic effect.

### 2.2. Clinical Investigation

**Figure 2:**
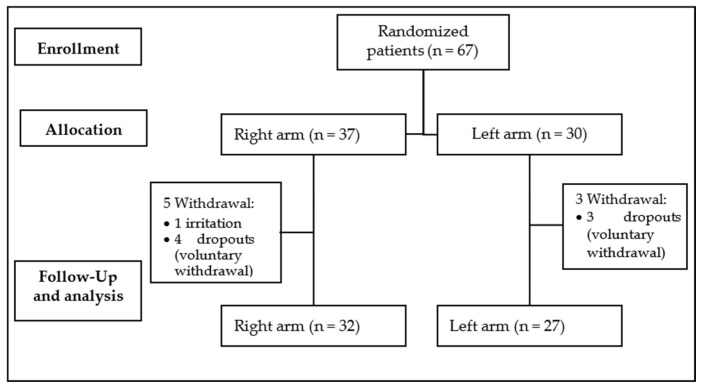
Diagram CONSORT profile.

In the clinical trial, from January 2023 to April 2024, 67 patients were enrolled and randomly divided in two groups, with 37 in the right arm group and 30 in the left arm group. Eight patients dropped out of the study, one of them due to irritation; the rest decided voluntarily not to continue the study. Therefore, the evaluated population included 59 patients, as showed in Figure 2.

The baseline characteristics of the patients are shown in Table 3. 55.9% of patients were female. 64.4% of patients had phototype II. 78% of patients applied creams one to three times per week. 45.8% of patients applied the emulgel to test on the left arm. Only 10% of patients did not need any additional treatment; the rest of the patients continued with their conventional treatment. 21% of patients had no comorbidities such as prurigo nodularis, asthma, allergies, contact dermatitis, conjunctivitis or rhinitis. 69% of patients had occasional exposure to the sun.

**Figure 3:**
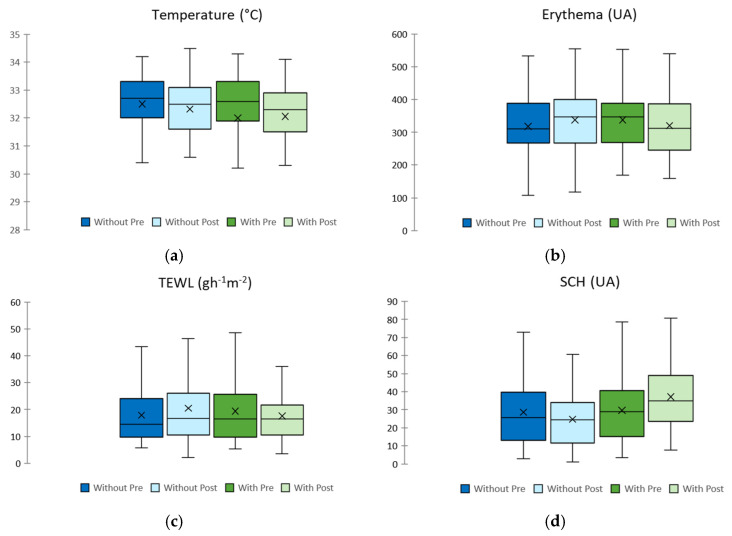

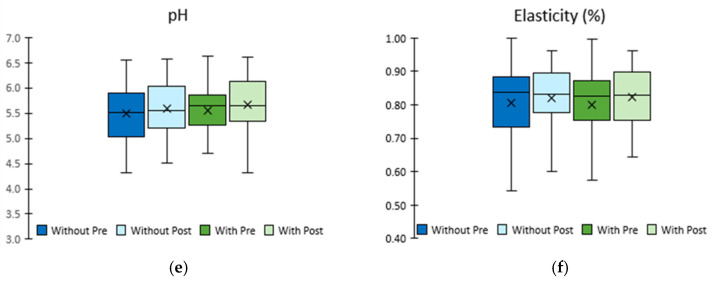
Changes in homeostasis parameters in eczematous areas. (**a**)Temperature assessment, (**b**) Erythema assessment, (**c**) TEWL assessment, (**d**) SCH assessment, (**e**) pH assessment, (**f**) elasticity assessment.

Table 4 shows the observed changes in homeostasis parameters in healthy and eczematous areas when emulgel was used in the experimental arm compared to the control arm. Figure 3 shows a bar graph comparing each homeostasis parameter in the intervention and control arms before and after the application of emulgel.

**Figure 4:**
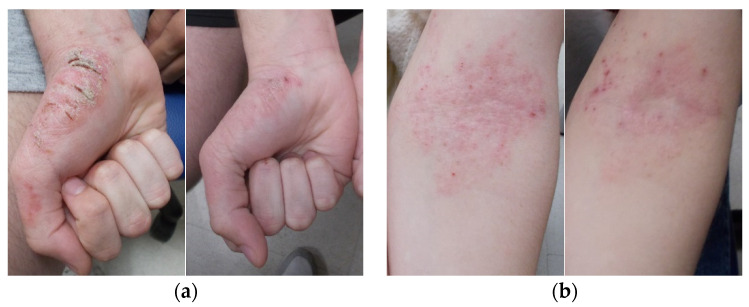
Images of areas affected by AD before and after treatment: (**a**) patient’s hand;(**b**) patient’s elbow flexure.

The results of our study show that there is an improvement in the epidermal barrier function after 10 days of emulgel application, as shown in Figure 4.

The results obtained suggest that the combination of the key components of emulgel is responsible for the improvement in the skin barrier. In vitro studies have shown that EGF reduces TEWL, epidermal thickness, AD inflammation and total and allergen-specific immunoglobulin E (IgE) [25]. Glycerol, present in the stratum corneum as a natural endogenous humectant [38], inhibits the transition of the intercellular lamellar lipids from a liquid to a solid phase and accelerates the recovery of the skin barrier when disrupted [39]. HA is essential for maintaining the stratum corneum structure and epidermal barrier function [38]. Aloe vera increases the SCH and decreases the TEWL, resulting in an improvement in the physiological function of the skin [40]. Calendula officinalis exhibits angiogenic and fibroblastic activity, which has a positive effect on the proliferative stages of skin wound healing [41], and anti-inflammatory and anti-pruritic properties in induced AD-like mice models [34].

Regarding the temperature, our study shows no significant change after 10 days of emulgel application in the eczematous area, but it shows changes in the healthy area (*p* = 0.017), which is consistent with the patients’ opinion about the sensation of freshness provided by the emulgel. The skin temperature measured is considered within the normal forearm temperature range as described by Lee et al., who considered a normal range between 31.7 and 33.5 °C [42].

Erythema showed a decrease after 10 days of emulgel application in the eczematous area, and it is considered statistically significant (*p* = 0.008) when compared with the forearm that remained without cream application. Erythema is considered an indicator of irritation. The reduction in this parameter allows us to conclude that the emulgel is not irritating to the skin, and could also have an anti-inflammatory effect, which could be due to *Calendula officinalis* [43,44,45] and Aloe vera [46,47]. In addition, it can be seen that when the emulgel is not applied, there is a statistically significant (*p* = 0.02) worsening of erythema in the control arm within 10 days, so it could be said that the emulgel not only improves redness and swelling but also prevents their worsening.

TEWL is one of the main parameters used to assess the skin barrier function, and it shows a decrease after 10 days of emulgel application, which tends to be statistically significant (*p* = 0.006) when compared to the forearm without cream application in the eczematous area. The normal range for TEWL values is between 1 and 25 g/m^2^/h, and TEWL values above this range indicate an alteration in the skin barrier function [48]. The TEWL results obtained in our study are within this normal range.

SCH is a relevant parameter to assess the skin barrier function. Lower values have been associated with epidermal barrier disorders such as atopic dermatitis [49]. In our study, an increase in SCH was observed and there was a significant difference after 10 days of emulgel application (*p* < 0.001), and between both forearms in eczema and healthy areas. SCH is increased in healthy skin when treated with our emulgel (*p* < 0.000), so according to atopic guidelines, this product can be used as a daily treatment on healthy skin.

Skin pH shows no differences between before and after emulgel application and between both forearms. It is an important parameter to determine the skin barrier function, as an acidic pH ensures the lipid organization and metabolism necessary for the proper activity of the stratum corneum, and we observed in our study that the measured skin pH is within the values considered to be the normal range to protect the skin against damaging exogenous factors [48]. Skin pH is elevated in different situations such as neonatal skin, sensitive skin, or inflammatory skin conditions such as atopic dermatitis and acne [50].

Elasticity is a parameter related to the biomechanical properties of the skin. In our study, it shows differences between before and after emulgel application in eczema (*p* = 0.026) and healthy areas (*p* = 0.017), but not between both forearms.

**Figure 5:**
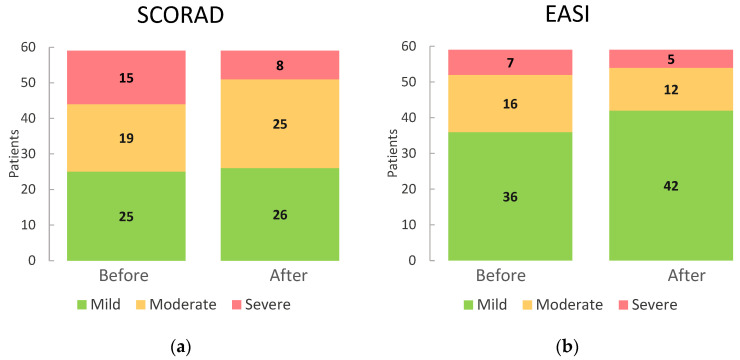
(**a**)Change in SCORAD before and after the use of emulgel. (**b**) Change in EASI before and after the use of emulgel.

Table 5 shows the observed changes in SCORAD and EASI values before and after using emulgel. Figure 5 shows a bar graph comparing SCORAD and EASI values before and after the application of emulgel.

Regarding the SCORAD value, our study shows that between the beginning and the end of treatment with emulgel, the number of severe patients decreased from 15 to 8, while the number of patients with mild AD increased from 25 to 26, so we can assume that there is an improvement in the overall clinical condition of the patients and a decrease in the severity of the disease.

In terms of the EASI index, there was also an improvement in the general condition of the patients, as the number of severe cases decreased from 7 to 5 and the number of moderate cases decreased from 16 to 12, while the number of patients with mild AD increased from 36 to 42.

**Figure 6:**
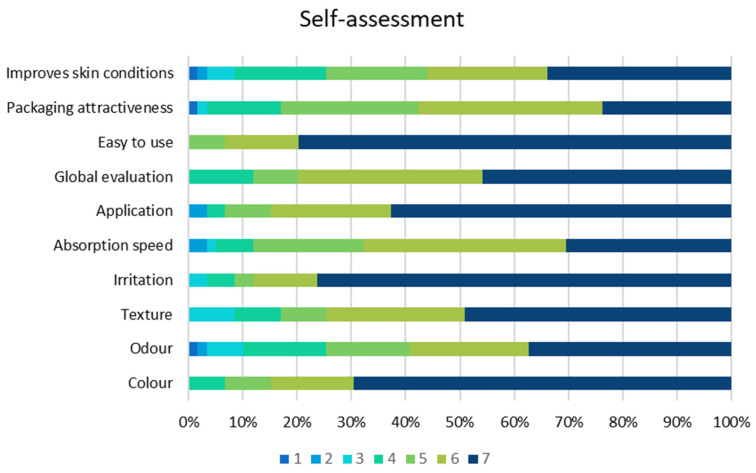
Self-assessment by patients.

All participants rated the tolerability of the cream very positively in terms of texture, colour, odour, absorption, irritation, application, ease of use, packaging and improvement in skin condition. No adverse effects were reported except for one case of minor irritation. The cream used in the study was safe, effective and very well tolerated, as shown in Figure 6.

Our study has some limitations. All patients included in the study were adolescents >14 years old and adults so more research is needed to evaluate the effects of our product on children, as AD is a disease with a high prevalence in this age group. Further studies comparing the developed emulgel with conventional drugs should be carried out to evaluate the real effects of each treatment. Our study lasted 10 days; longer studies should be carried out with a larger sample including patients of different severities. Blinding should be considered in future studies to avoid the risk of bias.

## 3. Conclusions

Treatment of AD with an emulgel based on sodium hyaluronate, glycerol, grape seed oil, *Calendula officinalis*, *Aloe vera*, and EGF as active ingredients in addition to conventional treatments can be a valuable option for dermatologists. Within the limitations of this study, the emulgel improved homeostasis parameters such as temperature, TEWL and stratum corneum hydration, and improved SCORAD and EASI scores after 10 days of treatment. Further long-term studies with larger samples and children as patients are needed.

## 4. Materials and Methods

### 4.1. Preparation of Emulgel

An emulgel based on sodium hyaluronate, glycerol, grape seed oil, *Calendula officinalis*, *Aloe vera*, and EGF as active ingredients was developed. The formulation included emollient, humectant, surfactant, antioxidant and buffering components, soluble in both oil and aqueous phases, and also included carbomer as a gelling agent to obtain the emulgel as described in Table 6.

The obtained emulgel presented the following physicochemical characteristics:

1. Appearance: Easy to apply and absorb light green emulgel
2. Infrared spectrum: similar to the reference sample (the infrared spectrum correlation coefficient compares the spectrum of the sample at t = 0 with the spectrum of the corresponding sample, taking a value of 1 for the first sample and should be greater than 0.95 for the compared ones).

**Figure 7:**
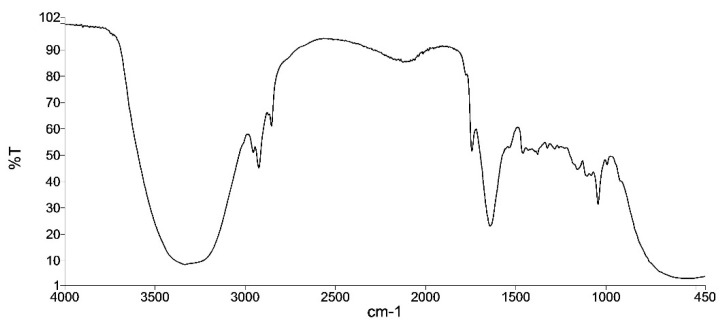
Emulgel infrared spectrum.

The infrared spectrum is divided into three main regions: the bond-stretching region, the bond-bending region and the fingerprint region. Our emulgel infrared spectrum is shown in Figure 7; it lies between the wavelengths of 450 and 4500 cm^−1^, but no specific component can be easily identified.

1. pH: 5.5–6.5
2. Relative density: 0.95–1.05

The infrared spectrum, pH and relative density were performed according to Eu. Ph 10th Edition, Volume 1, 2019. The appearance was subjectively evaluated.

And the following microbiological properties were evaluated according to ISO 17516:2014 [51]:

1. Total Aerobic Microbial Count (TAMC): <1000 cfu/g or mL
2. Total Yeast and Mold Count (TYMC): <100 cfu/g or mL
3. Absence of pathogens: absence/g or mL
  *3.1.*  *Candida albicans*
  *3.2.*  *Pseudomonas aeruginosa*
  *3.3.*  *Staphylococcus aureus*
  *3.4.*  *Escherichia coli*

The stability of the developed formulation was evaluated according to the International Conference of Harmonisation (ICH) guidelines Q1A [52] and ISO/TR 18811:2018 [53]. A study under accelerated conditions (40 ± 2 °C and 75 ± 5% relative humidity) and a study under long-term conditions (25 ± 2 °C and 60 ± 5% relative humidity) were carried out to ensure the stability of the formulation not only during the investigation, but also in the longer term to evaluate the shelf life of the formulation. No differences were observed in the following parameters tested: appearance, pH, infrared spectrum, TAMC and TYMC, up to 6 months under accelerated conditions and 48 months under long-term conditions, as shown in Table 7 and Table 8.

### 4.2. Biocompatibility Tests

The safety of the developed emulgel was tested by performing biocompatibility studies, such as irritation, sensitization and cytotoxicity, with equivalent formulations sharing the main components (sodium hyaluronate, glycerol, EGF, *Calendula officinalis*, and *Aloe vera*) and their concentrations or even higher in the tested product. All the tests were conducted in accordance with ISO 10993-1:2009 [54] requirements. Animal welfare was ensured in accordance with ISO 10993-2:2006 [55] guidelines.

#### 4.2.1. Skin Irritation

Healthy adult albino rabbits were used for the irritation test and the study product was applied to them together with two controls, one positive and one negative according to the protocols described in ISO 10993-10:2010 [56]. For the evaluation of the results, the application sites were examined with binocular loupes (3X).

#### 4.2.2. Sensitization Test

Healthy adult guinea pigs were used for the sensitization test and the study product, and a negative control was applied according to the protocols described in ISO 10993-10:2010. The application sites were examined for visible skin reactions.

#### 4.2.3. Cytotoxicity Test

The L929 mouse cell line was used as the test subject for the cytotoxicity test, as recommended in ISO 10993-5:2009 [57] as representative of the mammalian system. Cells were seeded on the plates and the test item extract and controls were added and incubated. Cytotoxicity was measured by the WST-1 cell viability assay (colorimetric).

### 4.3. Clinical Investigation

#### 4.3.1. Study Design

A single-centre randomised self-controlled clinical trial was conducted at the Hospital Universitario Virgen de las Nieves in Granada (Spain).

The study protocol was developed in accordance with the Declaration of Helsinki and ISO 14155:2020 [58], and ethical clearance was obtained from the Andalusian Biomedical Research Ethics Portal (Project identification code 2327-M1-21) and from the Spanish Agency for Medicines and Medical Devices (AEMPS). The procedures performed were non-invasive.

Patients were treated with the test emulgel in addition to conventional treatment (topical/systemic corticosteroids/monoclonal antibody/antibiotic) in one arm and no repair cream in the other arm as a control for 10 days. Patients received their conventional treatment and did not apply any other moisturiser/repair cream. Patients were randomised to the left or right intervention group arm.

All participants were assessed at the baseline and instructed to apply the emulgel daily to the area of the upper arm indicated by the physician at the first visit and no other moisturiser to the other arm to assess the effect of the emulgel. All participants received the following skin care instructions: no swimming in chlorinated water, no bleach baths, no antibiotic creams for 7 days prior to the study visits; no moisturisers, bathing or showering for 24 h prior to the study visit. Use SYNDET soap.

(1) Control arm: Patients followed their doctor’s prescribed treatment for AD.

(2) Experimental arm: Patients followed the treatment prescribed by their physician and applied the emulgel twice daily to the area to be evaluated.

#### 4.3.2. Participants and Procedures

Patients of both sexes, older than 14 years, as the minimum age to be treated in adult consultation, treated in adult outpatient clinic, with AD according to Hanifin and Rajka’s criteria, subject with mild to moderate AD (SCORAD between 1 and 40) and who are amenable to conventional treatment, whose dermatitis is present in the upper artery areas and whose dermatitis affects less than 15% of the body surface area. Patients with skin infections or infestations, HIV, cancer of any type, proven immunosuppression, concomitant application of another moisturiser, with the face as the affected area were excluded from the study.

#### 4.3.3. Randomization

The random sequence was generated using EXCEL prior to patient enrolment. Patients were allocated to each group by simple randomisation. A table of random numbers without repetition was generated. In order of arrival, patients were assigned to the left intervention and right control groups with even numbers and to the right intervention and left control groups with odd numbers.

#### 4.3.4. Outcomes

The primary objective was to evaluate the efficacy of the emulgel in combination with conventional physician-directed therapy in patients with AD, assessed by measuring homeostasis parameters, SCORAD and EASI values. The secondary objective of the study was to determine the subjective opinion of the patients included in the study, its acceptability, absorption, comfort of use and tolerability.

The following homeostasis parameters related to the epidermal barrier function were measured to evaluate the efficacy of the emulgel to treat AD:

Transepidermal water loss (TEWL) indicates the water that diffuses from the dermis and epidermis through the stratum corneum; when elevated, it is associated with skin barrier dysfunction [59]. It is measured with a Tewameter TM300 (Skin analyser Microcaya S.L., Bilbao, Spain).

Skin temperature as an indicator of skin microcirculation, and when elevated it is associated with skin barrier dysfunction [60]. It is measured using an ST 500 skin thermometer (Skin analyser Microcaya S.L., Bilbao, Spain).

Erythema appears when the skin is exposed to irritants such as chemicals, detergents, allergens, or UV rays [61]. The erythema index is measured with an MX 18 mexameter (Skin analyser Microcaya S.L., Bilbao, Spain).

Reduced stratum corneum hydration (SCH) is associated with skin barrier dysfunction and alters the structure of intercellular lipids [59,62]. It is measured with a CM 825 corneometer (Skin analyser Microcaya S.L., Bilbao, Spain).

An acidic pH is required in the stratum corneum to ensure lipid organization and lipid metabolism [63]. Skin pH is measured with a PH 905 skin pH meter (Skin analyser Microcaya S.L., Bilbao, Spain).

Skin elasticity is measured with a Cutometer dual MPA 580 (Skin analyser Microcaya S.L., Bilbao, Spain). Elasticity acts as an indicator of the degradation of elastic fibres in the skin [64].

Measurements were carried out using previously described probes adapted to an MPA 580 multiprobe system (MPA COURAGE+KHAZAKA electronicGmbH, MICRO-CAYA, S.L, Bilbao, Spain).

The SCORAD (SCORing Atopic Dermatitis) is a scale for assessing the severity of signs and symptoms of AD, such as the degree of erythema, oedema, papules, exudate, excoriation, lichenification, pain and xerosis, as well as pruritus and sleep disturbance according to the VAS scale [65]. The severity is classified according to the obtained score in SCORAD, mild (<25), moderate (25–50) and severe (>50) [66].

The EASI (Eczema Area and Severity Index) is another scale that includes an assessment of the extent of the disease and the percentage of the body surface area affected in patients with AD. The severity is classified according to the obtained score in EASI, mild (≤7), moderate (7.1–21) and severe (>21) [67].

The EASI-SCORAD calculator application (Sanofi Genzyme) is used to determine SCORAD and EASI.

#### 4.3.5. Statistical Analyses

Statistical analyses were performed using an SPSS package (IBM Corp. Released 2019. IBM SPSS Statistics for Windows, Version 26.0. Armonk, NY, USA: IBM Corp). Variables were compiled in a database and statistically analysed to determine the significance of the results. Changes in the study area between treatment sessions were assessed. Qualitative variables were presented as absolute (relative) frequencies and quantitative variables as mean and standard deviations. Normality of variables was tested using the Kolmogorov–Smirnov test and the Shapiro–Wilk test. Categorical data were compared using the chi-squared test.

The Student’s *t*-test was used to compare continuous variables in independent samples, and the Student’s *t*-test for paired samples was used to compare variables in the same individual. Statistical significance was considered when *p* < 0.05 with two tails.

## Figures and Tables

**Table 1:** Scores for skin irritation test for test and control samples.

Samples	Primary Irritation Score			Primary Irritation Index
	Rabbit ID1	Rabbit ID2	Rabbit ID3	
Study product	0	0	0	0
Positive control	4.00	5.33	4.33	4.55
Negative control	0	0	0	0

**Table 2:** Observation values for sensitization test.

Groups	Groups Mean Topical Induction Phase—ChallengePhase	Score
Male test	0.8	0.7
Female test	0.6	
Negative control	0.4	0.4

**Table 3:** Baseline characteristics of AD patients. Continuous variables expressed as mean (SD) and nominal variables as absolute frequencies (relative).

Characteristics	Mean/No	SD/%
Women	33	55.9
Age (years)	31.8	16.20
Phototype: I II III IV	5 38 14 2	8.5 64.4 23.7 3.4
Cream application: <1/week 1–3/week 4–6/week >6/week	8 46 1 4	13.6 78.0 1.7 6.8
Experimental arm: Left Right	27 32	45.8 54.2
Treatment: Topical Cyclosporines Biological None	26 7 20 6	44.1 11.9 33.9 10.2
Comorbidities: Prurigo nodularis Asthma Allergies Contact dermatitis Conjunctivitis Rhinitis None	4 27 25 16 6 7 22	4 25 23 15 6 7 21
Solar exposure: Never Occasionally Frequently	13 41 5	22 69 8

**Table 4:** Changes in homeostasis parameters in healthy and eczematous areas (n = 59).

**Homeostasis** **Parameter**	**Healthy Zone**								
	**Forearm without Emulgel**				**Forearm with Emulgel**				*p* *
	**before**	**after**	**Dif.**	***p* ^1^**	**before**	**after**	**Dif.**	***p* ^2^**	
Temperature (°C)	32.00 ± 0.13	31.71 ± 0.14	−0.29 ± 0.14	0.050	31.98 ± 0.13	31.64 ± 0.15	−0.34 ± 0.14	0.017	0.509
Erythema (AU)	210.81 ± 8.80	216.8 ± 9.58	6.08 ± 5.20	0.247	210.49 ± 10.17	215.35 ± 10.17	4.86 ± 6.80	0.478	0.868
TEWL (g·m^−2^·h^−1^)	11.64 ± 0.78	11.05 ± 0.72	−0.59 ± 0.65	0.372	11.48 ± 0.72	10.81 ± 0.69	0.67 ± 0.65	0.308	0.894
SCH (AU)	35.79 ± 1.54	33.32 ± 1.62	−2.47 ± 1.30	0.062	35.65 ± 1.59	44.02 ± 1.61	8.37 ± 1.55	<0.000	<0.000
pH ^#^	5.40 ± 0.07	5.47 ± 0.07	0.07 ± 0.11	0.522	5.42 ± 0.07	5.51 ± 0.07	0.09 ± 0.09	0.341	0.748
Elasticity (%)	0.828 ± 0.011	0.843 ± 0.010	0.015 ± 0.006	0.024	0.817 ± 0.011	0.835 ± 0.011	0.019 ± 0.007	0.017	0.702
**Homeostasis** **Parameter**	**Eczema Zone**								
	**Forearm without Emulgel**				**Forearm with Emulgel**				*p* *
	**before**	**after**	**Dif.**	***p* ^1^**	**before**	**after**	**Dif.**	***p* ^2^**	
Temperature (°C)	32.51 ± 0.12	32.32 ± 0.16	−0.18 ± 0.15	0.229	31.99 ± 0.51	32.06 ± 0.18	0.07 ± 0.54	0.904	0.631
Erythema (AU)	318.00 ± 10.73	337.60 ± 11.37	19 60 ± 8.16	0.020	337.74 ± 11.78	320.55 ± 11.79	−17.18 ± 12.18	0.164	0.008
TEWL (g·m^−2^·h^−1^)	17.90 ± 1.27	20.51 ± 1.69	2.61 ± 1.74	0.140	19.41 ± 1.46	17.63 ± 1.20	−1.77 ± 1.50	0.243	0.006
SCH (AU)	28.67 ± 2.32	24.74 ± 2.02	−3.93 ± 2.00	0.054	29.64 ± 2.29	37.15 ± 2.23	7.51 ± 2.16	0.001	<0.000
pH ^#^	5.48 ± 0.07	5.58 ± 0.06	0.10 ± 0.09	0.245	5.55 ± 0.06	5.66 ± 0.07	0.11 ± 0.09	0.229	0.862
Elasticity (%)	0.807 ± 0.013	0.819 ± 0.013	0.012 ± 0.013	0.373	0.799 ± 0.013	0.824 ± 0.011	0.025 ± 0.010	0.026	0.411

AU, arbitrary units; SCH, Stratum Corneum Hydration; TEWL, Transepidermal Water Loss. ^#^ Calculated values for 56 patients. ^1^ *p*-value after using Student’s *t*-test for paired samples to compare baseline and end-of-follow-up epidermal barrier function parameters in the arm that did not receive repair cream. ^2^ *p*-value calculated using Student’s *t*-test for paired samples to compare baseline and end-of-treatment epidermal barrier function parameters in the repair cream arm. * *p* value after using Student’s *t*-test for paired samples to compare changes in epidermal barrier function parameters between the two arms.

**Table 5:** Changes in SCORAD and EASI values before and after using emulgel (n = 59).

	Before Emulgel	After Emulgel
SCORAD:		
Mild (<25)	25 (42.4%)	26 (44.1%)
Moderate (25–50)	19 (32.2%)	25 (42.4%)
Severe (50–103)	15 (25.4%)	8 (13.6%)
Total	59 (100.0%)	59 (100.0%)
EASI:		
Mild (≤7)	36 (61.0%)	42 (71.2%)
Moderate (7.1–21)	16 (27.1%)	12 (20.3%)
Severe (>21.1)	7 (11.9%)	5 (8.5%)
Total	59 (100.0%)	59 (100.0%)

**Table 6:** Ingredients, concentration, function and formula phase (active ingredients in bold).

Function	Components	Concentration (*w*/*v*)	Emulsion		Gel
			Oily Phase	Aqueous Phase	
**Emollient, humectant, moisturizer**	**Glycerol** **Sodium hyaluronate** Panthenol **Aloe barbadensis leaf juice** **Calendula officinalis flower extract** Propylene glycol Zinc gluconate	**5.0%** **1.0%** >0.1–≤1.0% **1.0%** **1.8%** >1.0–≤5.0% ≤0.1%		x	
	**Grape seed oil** Allantoin Caprylic/capric triglyceride C13–14isoparaffin Polyacrylamide	**3.0%** >0.1–≤1.0% >1.0–≤5.0% >0.1–≤1.0% >1.0–≤5.0%	x		
Antioxidant	Tocopheryl acetate Tocopherol Pentaerythrityl tetra-di-T-butyl hydroxyhydrocinnamate	≤0.1% ≤0.1% ≤0.1%	x		
Solvent	Aqua	>50.0–≤75.0%		x	
Chelating	Disodium EDTA	≤0.1%		x	
Colorant	CI42090	≤0.1%		x	
Growth Factor	Epidermal Growth Factor *	**0.00001%**	x		
Surfactant	PEG-18 castor oil dioleate PEG/PPG-4/12 dimethicone Laureth-7	>0.1–≤1.0% >0.1–≤1.0% >0.1–≤1.0%	x		
Preservative	Potassium sorbate Sodium benzoate	≤0.1% ≤0.1%		x	
	BHT	≤0.1%	x		
Buffer	Citric acid	≤0.1%		x	
	Triisopropanolamine	≤0.1%	x		
**Gelling**	Carbomer	>0.1–≤1.0%			x

x indicates the phase in which the ingredient has been incorporated. * As EGF is very unstable, it was formulated using a patented solution (US Patent No. US 11,147,882 B2) to stabilise it and allow it to be formulated in an emulsion containing an aqueous phase.

**Table 7:** Accelerated condition study results.

Parameter	T = 0	T = 1 Month	T = 3 Months	T = 6 Months
Appearance	✓	✓	✓	✓
pH	5.7	5.4	5.3	5.8
Infrared spectrum Correlation coefficient	1	0.992	0.990	0.954
TAMC	<100 cfu/mL	-	<100 cfu/mL	-
TYMC	<100 cfu/mL	-	<100 cfu/mL	-

✓: complies with appearance specifications.

**Table 8:** Long-term condition study results.

Parameter	T = 0	T = 3 Months	T = 6 Months	T = 9 Months	T = 12 Months	T = 18 Months	T = 24 Months	T = 36 Months	T = 48 Months	T = 60 Months
Appearance	✓	✓	✓	✓	✓	✓	✓	✓	✓	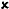 *
pH	5.7	5.3	5.2	5.3	5.3	5.3	5.3	5.3	5.3	5.2
Infrared spectrum	1	0.981	0.970	0.958	0.995	0.995	0.993	0.988	0.982	0.743
TAMC	<100 cfu/mL	-	-	-	-	-	<100 cfu/mL	-	-	*
TYMC	<100 cfu/mL	-	-	-	-	-	<100 cfu/mL	-	-	*

✓: complies with appearance specifications, 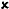: does not comply with appearance specifications * TAMC and TYMC not carried out due to the change in appearance, yellowish cream, and infrared spectrum coefficient correlation out of specifications.

## Data Availability

The data presented in this study are available on request from the corresponding author. The data are not publicly available due to the privacy of the patients who assisted in the research.
